# Ocular Delivery System for Propranolol Hydrochloride Based on Nanostructured Lipid Carrier

**DOI:** 10.3390/scipharm86020016

**Published:** 2018-04-20

**Authors:** Behzad Sharif Makhmal Zadeh, Hassan Niro, Fakher Rahim, Golbarg Esfahani

**Affiliations:** 1 Nanotechnology Research Center, School of Pharmacy, Ahvaz Jundishapur University of Medical Sciences, 6135715794 Ahvaz, Iran; 2 Department of Pharmaceutics, School of Pharmacy, Ahvaz Jundishapur University of Medical Sciences, 6135715794 Ahvaz, Iran; hnfarmad@yahoo.com (H.N.); golbarg_isfahani@yahoo.com (G.E.); 3 Research Center of Thalassemia & Hemoglobinopathy, Ahvaz Jundishapur University of Medical Ciences, 6135715794 Ahvaz, Iran; Bioinfo2003@gmail.com

**Keywords:** ocular, drug delivery, permeability, propranolol hydrochloride, nanostructured lipid carrier

## Abstract

One drawback of traditional forms of medical ocular dosage is drug dilution by tear; moreover, drugs are rapidly drained away from pre-corneal cavity by tear flow and lacrimo-nasal drainage. Prolonging contact time with different strategies and mucoadhesive vehicles will help to continuously deliver drugs to the eyes. For this study, we prepared and evaluated the effects of a nanostructure lipid carrier (NLC) on propranolol hydrochloride as a hydrophilic drug model for rabbit corneal permeation. Propranolol hydrochloride NLC was prepared using cold homogenization. The lipid was melted, then the drug and surfactant were dispersed and stirred into the melted lipid. This fused lipid phase was scattered in aqueous solution containing the cosurfactant at 4 °C and then homogenized. We evaluated particle size, drug loading, drug release, and NLC permeability through rabbit cornea as well as the formula’s effect on the cornea. Our results show that drug loading efficiency depended on the surfactant/lipid ratio (S/L) and the percentages of liquid lipid and Transcutol (Gattefosse, Saint-Priest, France) (as solubilizer). Drug release data were evaluated with the Higuchi model and a significant correlation was shown between the S/L ratio and the amount of drug released after 4 and 48 h. NLC formulations improved propranolol hydrochloride permeation. We conclude that the effect of the NLC formulations was due to mucoadhesive and film forming properties.

## 1. Introduction

Topical drug delivery is a convenient mode of drug administration for ocular diseases. Yet, bioavailability through traditional ocular dosage forms, such as eye drops, is very poor. Several factors—such as pH, lachrymal secretion, blinking, tear flow, and epithelial barriers—influence ocular drug bioavailability [1]. Drainage of most of the drug into the nasolacrimal duct within a few minutes and systemic absorption via conjunctional circulation decrease ocular drug concentration. To provide effective ocular drug concentration, repeated dosing may be required, which may lead to patient noncompliance. Prolongation of pre-corneal residence time is needed to improve the drug bioavailability of topically administered ocular drugs [2]. Additionally, eye drops are not a suitable dosage form for all purposes––for instance, delivery to the posterior segment––and alternative drug delivery systems are needed to achieve effective concentration in target sites [3,4]. A perfect ocular dosage form should be safe and provide selective targeting to the ocular tissue and prolonged delivery with minimal systemic effect [5].

Nanoparticle-based products demonstrate three capabilities, including enhanced drug permeation, sustained and controlled drug delivery, and targeted drug delivery. Encapsulation of drugs in these colloidal carriers improves therapeutic effectiveness in comparison with traditional ocular dosage forms (such as eye drops), as has been shown by studies of different nanostructured carriers [6,7,8,9]. The effectiveness of nanoparticles as ocular delivery systems depends on many factors, including increased pre-corneal retention time, the method of preparation, pre-corneal biodegradation, optimization of the lipophilic–hydrophilic properties of the carrier drug system, and the effects of nanoparticles on corneal structure [10].

Nanostructured lipid carriers (NLC) are prepared using solid and liquid lipids, surfactant, and water and range in size from 50 to 1000 nm [11]. Previous studies have reported the use of solid lipid nanoparticles as ocular delivery systems [12]. NLC interaction with the corneal mucosa based on biocompatibility and mucoadhesive properties increases the drug’s corneal contact time and improves the ocular bioavailability [13]. The effect of solid lipid nanoparticles (SLN) on tobramycin ocular delivery was reported previously [14]. Furthermore, SLN significantly increases drug bioavailability in the aqueous humor. In another study, poorly water-soluble drugs (such as hydrocortisone, estradiol, and pilocarpine) were incorporated into SLN [15] and corneal permeability was evaluated. The study demonstrated prolonged drug release in all formulations. Ex vivo studies of trans-corneal permeation in animal models have been used to characterize passive cornea permeation. Although permeation studies in such models neglect the complications of tear flow, tear drainage, and blinking, they have provided information about targeting similar molecules from the same pharmacological class [16]. The aim of the present study was the preparation of the NLC and evaluation of rabbit corneal permeation of propranolol hydrochloride as a beta-blocker agent.

## 2. Materials and Methods

### 2.1. Materials

Propranolol hydrochloride was obtained as a gift from Daropakhesh Pharmaceutical Company (Tehran, Iran). Oleic acid, Span 20, and Tween 80 were purchased from Merck (Berlin, Germany). Cellulose acetate membrane (12 KDa) was obtained from Sigma Aldrich (St. Louis, MO, USA) (Corning, NY, USA). Male rabbits weighting 2.5–3 kg were purchased from Animal House (Ahvaz Jundishapur University of Medical Science, Ahvaz, Iran). Compritol ATO 888 and Transcutol P were gifted by Gattefosse (Faratin Company, Tehran, Iran). All other chemicals were analytical grade.

### 2.2. Methods

#### 2.2.1. Preparation of Drug-Loaded Nanostructure Lipid Carrier (NLC)

Propranolol hydrochloride NLC was prepared by cold homogenization according to the procedure described in Lidtke et al. [17]. Lipids (15% *w*/*v*) including Compritol ATO 888 (solid lipid) and oleic acid (liquid lipid) were melted, then 0.5% drug, 1.5–3% Tween 80+ Span 20 (1:1) as surfactants and 0–1% Transcutol as solubilizer were dispersed and stirred into the melted lipid, followed by sonication for 2 min. This fused lipid phase was cooled rapidly by placing in dry ice. Then, the drug-containing solid lipid was pulverized to microparticles by mortar milling. Microparticles were scattered in aqueous solution containing 2% propylene glycol as cosolvent at 4 °C (the final volume was 50 mL). Then, the suspension was passed through a homogenizer for three cycles of 20 s each at 2000 bar.

#### 2.2.2. Particle Size Measurement

Mean particle size and the polydispersity index of propranolol-hydrochloride-loaded NLCs were calculated by laser light diffractometry using a Malvern Mastersizer SM 2000 K (Malvern Instruments, Malvern, UK). Samples were prepared by dispersing NLCs with a sufficient amount of water, stirred, and followed by sonication for 2 min.

#### 2.2.3. Entrapment Efficiency (EE%) and Loading Capacity (LC%) Determination

The EE% and LC% determined by high pressure liquid chromatography (HPLC) (C18 column with 25 cm length and 4 µm particle diameter, phosphate buffer: acetonitril (50:50) with 1 mL/min flow rate as mobile phase and ultra violet (UV) at 280 nm as detector). For this purpose, using an indirect method, 2 mL of each NLC formulation were centrifuged at 20,000 rpm for 20 min and the amount of free drug in the supernatant was measured. The amount of encapsulated drug was calculated by subtracting the amount of free drug from that of the initial drug [14]. For the indirect method, the samples were centrifuged at 20,000 rpm for 20 min at 4 °C, and nanoparticles were separated from the suspending medium and dissolved in methanol and assayed by HPLC.EE% and LC% were calculated by the following equations:

EE% = (weight of initial drug − weight of free drug) × 100/(weight of free drug),(1)

(2) $$\mathrm{LC}\%=\frac{\mathrm{wt}\;\mathrm{of}\;\mathrm{drug}\;\mathrm{in}\;\mathrm{nanoparticles}}{\mathrm{wt}\;\mathrm{of}\;\mathrm{nanoparticles}}.$$

#### 2.2.4. Drug Release Profile of Nanostructure Lipid Carrier (NLC) Formulations

Drug release profiles were obtained by static diffusion cells with thermoregulated water jacket, with temperature maintained at 32 °C. A defined amount of the NLC formulation was centrifuged and the precipitate was collected and suspended in water. Five millilitres of NLC suspension was used as the donor phase and 33 mL of buffer phosphate (pH = 7) as receptor phase. Samples were collected in the determined intervals over 48 h [14]. The drug release mechanism was evaluated by fitting different models to the calculation correlation coefficient (R^2^).

#### 2.2.5. Drug Permeability through Isolated Rabbit Cornea

Male rabbit corneas were used for the permeation studies. The experiments were performed and approved (approval number: U-89042) in accordance with the guidelines for animal use of Ahvaz Jundishapur University of Medical Science (Iran), which were prepared by the National Academy of Sciences and published by the National Institutes of Health. Rabbits were sacrificed by intravenous ketamine injection and fresh corneas were excised, immediately weighed, and preserved in glutathione bicarbonate ringer (GBR) buffer. The corneal permeation studies were done using homemade Franz diffusion cells with horizontal design. In this experiment, the natural curvature of cornea was maintained. First, donor and receptor compartments were filled with GBR and tissue oxygenation performed using a mixture of 95% O_2_/5% CO_2_, bubbled across each compartment for 15 min. Thereafter, the donor phase was replaced with 2 mL of NLC containing 0.05 mg/mL propranolol hydrochloride, and 7 mL glutathione buffer was used to fill in receptor chambers [18]. Aqueous solution of propranolol hydrochloride with the same concentration as the test solution was used as a control. The corneas were applied into Franz cells within 0.5 h after excision. The cells were maintained at 34 °C. At time intervals of 30, 60, 120, 180 and 240 min, 1 mL of receptor phase was withdrawn and an equal amount of GBR buffer was added. The area for diffusion (*A*) was 0.7 cm^2^ and Equations (3) and (4) describe *P*_app_ (apparent permeability coefficient) and *J*_ss_ (flux or rate of permeation in steady state).

*P*_app_ = ∆*Q*/∆*t*·*C*_0_·*A*·60, (3)

*J*_ss_ = *C*_0_ × *P*_app._ (4)

In these equations, ΔQ/Δt is the steady state at the linear portion of the plot of amount of drug in the receptor chamber against time [19]. A is the diffusion area and *C*_0_ is the drug concentration loaded in donor.

#### 2.2.6. Effect of Nanostructure Lipid Carrier (NLC) on the Cornea

Increased hydration of corneal tissue indicates cornea damage by drug dosage forms, evaluated by different methods such as differential scanning calorimetric (DSC) [18]. Studies were performed with a Mettler DSC (Basel, Switzerland). Cornea samples equilibrated in GBR were immersed in NLC formulation for 1 h. Then, excess accelerant was removed and hermetically sealed into an aluminum pan. The thermograms were provided at a heating rate of 10 °C/min in the temperature range of −20 to 150 °C. The thermograms showed endothermic transition. Enthalpies (ΔH, J/g) were calculated using this equation [18]:

Δ*H* = peak area/sample weight. (5)

Results are presented as the effect of NLC formulation on transition temperature and enthalpy of the transition phase.

#### 2.2.7. Statistical Analysis

A 2^3^ factorial design was used to investigate the combined effect of three independent variables on the physicochemical and corneal permeation properties of different formulations that were prepared using cold homogenization. The effect of variables on formulation properties and corneal permeation was evaluated by Student’s unpaired *t*-test or one-way analysis of variance (ANOVA) (Minitab 16 software, Tehran, Iran) at *p* < 0.05.

## 3. Results

### 3.1. Formulation Components, Entrapment Efficiency (EE%) and Loading Capacity (LC%)

Different formulations’ properties are illustrated in Table 1. The experimental design was performed on the basis of full-factorial design, with three variables on two levels. The independent variables were surfactant/lipid ratio (S/L), liquid lipid percentage (%L) and Transcutol percentage (%T). These independent variables were selected based on preformulation study and previously reported research.

Factorial and variance analysis were performed in order to evaluate the impact of independent variables on EE%. The results illustrate that all variables had a significant impact on EE%; however, an increase in variables led to elevated drug loading. Since propranolol hydrochloride is a naturally hydrophilic compound, while the nanoparticles are lipophilic, the maximum loading capacity of 61% (Formulation (1)) is acceptable. On the other hand, the values of LC% were between 1.1% and 2.31%, with maximum and minimum values provided by Formulations (2) and (4), respectively.

### 3.2. Nanostructure Lipid Carrier(NLC) Particle Size Distribution

Table 2 summarizes the results regarding particle size and polydispersity index (PDI) for different formulations. The only independent variable that had a significant impact on particle size was %L: increasing the particle size showed a decreasing trend.

As mentioned above, surfactant concentration impact on particle size was not significant, a finding that shows consistency with some previous experiments, though it differs from others. For example, for nitrofurazone, the particle size increased with elevation of co-surfactant concentration [20], while the opposite results were obtained regarding chitosan-coated SLNs and repaglinide-loaded SLNs [21,22]. In addition, evaluation of PDI parameters demonstrates that particle size distribution follows a mono model in most formulations.

### 3.3. Drug Release from Lipid Nanoparticle Nanostructure Lipid Carrier (NLC)

**Figure 1:**
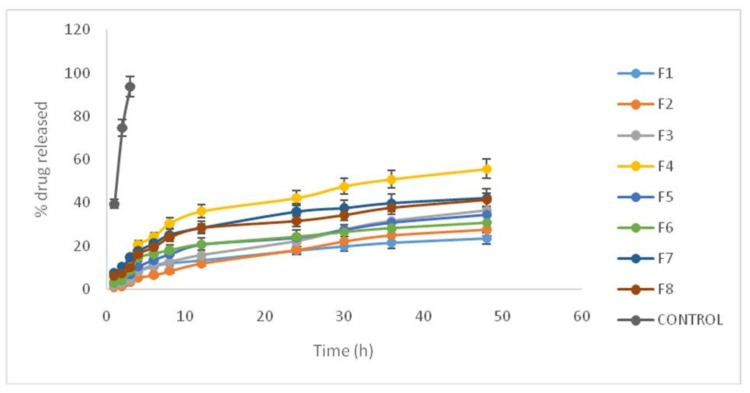
Propranolol hydrochloride release profile using different formulations.

The experiment was carried out in phosphate buffer with a pH of 7 and drug release was followed for48 h. In order to determine the effect of independent variables on drug release, the percentages of drug released after 4 h (R4) and 48 h (R48) were measured. R4 values measure rapid release of the component, whereas R48 quantifies the slow release rate (Table 3 and Figure 1). Aqueous solution of propranolol hydrochloride with the same concentration was used as control, and results showed that more than 98% of drug passed through membrane during 3 h. This means that permeation through acetate cellulose membrane was not the limiting step. 

As can be observed, the S/L ratio and %L percentage had a significant impact on drug release after 48 h. The results illustrate that an increase in the mentioned variables leads to a decreased drug release rate. These two variables were the reasons for increasing drug loading. Therefore, an increase in oleic acid and surfactant contents results in increasing propranolol hydrochloride loading and simultaneously causes a decrease in the drug’s release rate. Minimum amounts of R48 correspond to Formulation (1), where all three variables were at the highest level. On the other hand, Formulation (4), in which two variables (S/L and %L) were at minimum levels, had the highest value of R48. It seems that increasing the surfactant content promotes drug solubility in lipid matrix; consequently, drug loading in nanoparticles increases, whereas release rate decreases.

The drug release profile from NLC follows a two-step process: an initial rapid release with higher slope followed by a slow release with lower slope in the release profiles. Our results demonstrate that S/L ratio alone had a significant effect on R4 (*p* < 0.05); an increase in S/L ratio promotes drug loading after 4 h. However, R48 showed similar behavior. In addition, although liquid lipid percentage significantly affected R48, an increase in liquid lipid content resulted in a meaningful decrease at R48.This impact was not significant for R4 (*p* = 0.22). A comparison between the impact of %L on R4 and R48 indicates that oleic acid (OA) had no short-term impact on drug release, while the longer time period induced elevated drug loading in nanoparticles followed by a decrease in drug release rate. Therefore, S/L ratio and surfactant content play an important role in adjustment of optimum drug loading and release.

In a recent study, we used Compritol ATO 888 as the main solid lipid. It is composed of 64–72% mono- and diglycerides with a melting point of 71.1 °C. HLB 2 and its sustained release properties were previously reported [23]. In order to evaluate the drug release mechanism of NLC, we studied the release profile in three kinetic models: zero, first-order, and Higuchi models (Table 4).

Correlation coefficients and velocity constants in three situations were determined for all formulations. The results indicate that the Higuchi model was more consistent with the release profile. Accordingly, the main mechanism for control release of drug is diffusion, which strongly depends on the concentration gradient between the inside and outside environment of nanoparticles.

### 3.4. Nanoparticle Morphology

**Figure 2:**
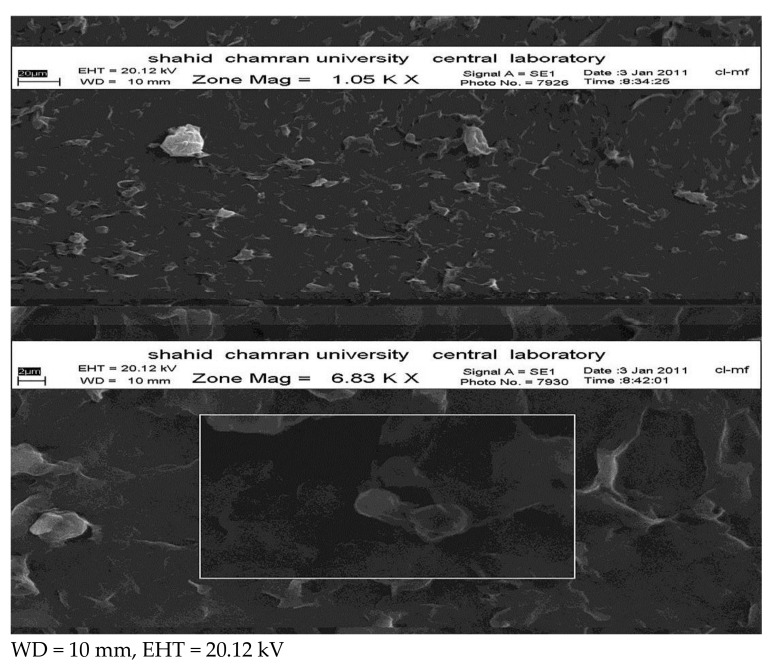
Scanning Electron Microscopy SEM imaging for lipid nanoparticles containing propranolol hydrochloride.

Figure 2 shows Scanning Electron Microscope SEM imaging for propranolol-hydrochloride-loaded NLCs. The figure above shows that the particles are mainly spherical and homogenized, on the basis of the solid solution pattern, while the impact layer surrounding the nanoparticles may account for the drug-enriched shell pattern, though it could be due to the topography of nanoparticles.

### 3.5. Nanostructure Lipid Carrier (NLC) Permeation through Rabbit Cornea

**Figure 3:**
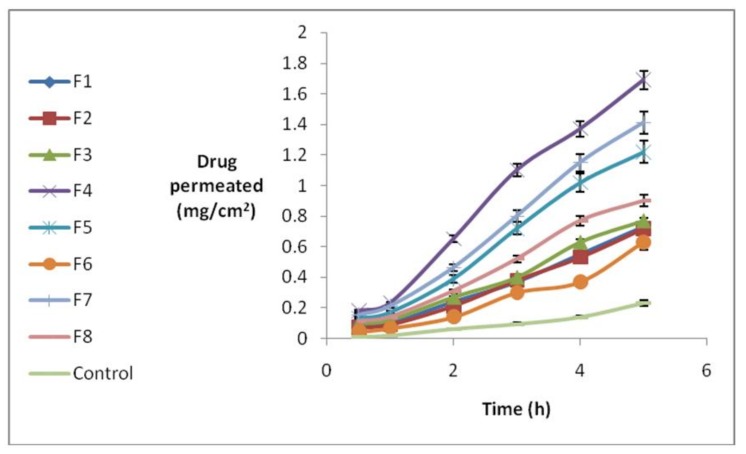
Permeated propranolol across cornea during a 5 h period (mean ± SD, *n* = 5).

In order to evaluate the effect of different formulations on propranolol-hydrochloride-NLC permeation, static diffusion cells and isolated rabbit cornea were used. The amount of permeated drug was measured hourly for 5 h. The results are shown in Figure 3.

Different permeation parameters were determined, including amount of drug permeating the surface area after 5 h (Q_5_) and the permeation rate, which can be obtained from the slope of drug amount against the time curve (*J*_ss_). *T*_lag_ was also determined by using the cumulative amount of permeated drug against time in a steady state. These parameters were measured on the basis of an infinite dose, considering sink condition. The results demonstrate that less than 10% of drug amount in the donor phase permeates through cornea, while the maximum concentration in the receiver phase was not more than 10% of drug saturation concentration in the receiver phase. Thus, sink condition and steady state were maintained. Table 5 summarizes different permeation parameters.

The maximum *Q*_5_ was 1.705 for Formulation (4), whereas the minimum content was 0.625 for Formulation (6). It should be mentioned that all formulations had significantly higher values of *Q*_5_ than the control (*p* = 0.001). It seems that only the S/L ratio had a significant but opposite effect on *Q*_5_. In other words, increased surfactant content led to an increase in loading and a significant decrease in *Q*_5_. We conclude that the effect of surfactant on *Q*_5_ was mainly due to drug loading. In addition, Transcutol impact on *Q*_5_ was not significant, perhaps due to its percentage in formulations. The effect of different concentrations of Transcutol as a permeation enhancer should be evaluated in another study. NLCs with a lipophilic nature and tendency toward cornea increased drug partitioning into cornea, causing promotion in *Q*_5_. The effect of independent variables on *J*_ss_ was similar to *Q*_5_, with the lowest and highest amounts of *J*_ss_ at 0.155 and 0.4 for Formulations (6) and (4), respectively. In addition, NLC formulations caused a significant *p* value increase in comparison to the control group (*p* = 0.001). According to our results, S/L ratio impact upon *J*_ss_ was significant; S/L increase leads to *J*_ss_ decrease.

T% and S/L ratio integration with *J*_ss_ is similar to *Q*_5_. Since *Q*_5_ and *J*_ss_ parameters were strongly influenced by drug concentration in the donor phase, and because of different loading efficiency in formulations, a negligible variation in *J*_ss_ and *Q*_5_ was observed due to different drug concentrations. The *P*_app_ in cornea was determined in order to normalize *J*_ss_ regarding drug concentration in the donor phase. The highest permeation coefficient was 0.068 for Formulation (4), while the lowest was 0.012 for Formulation (1). Comparison between permeation coefficients illustrates that although drug amounts in the NLC formulations’ donor phase were much higher than the control’s, all formulations had significant impact on P parameter.

### 3.6. Differential Scanning Calorimetric (DSC) of Rabbit Cornea

**Figure 4:**
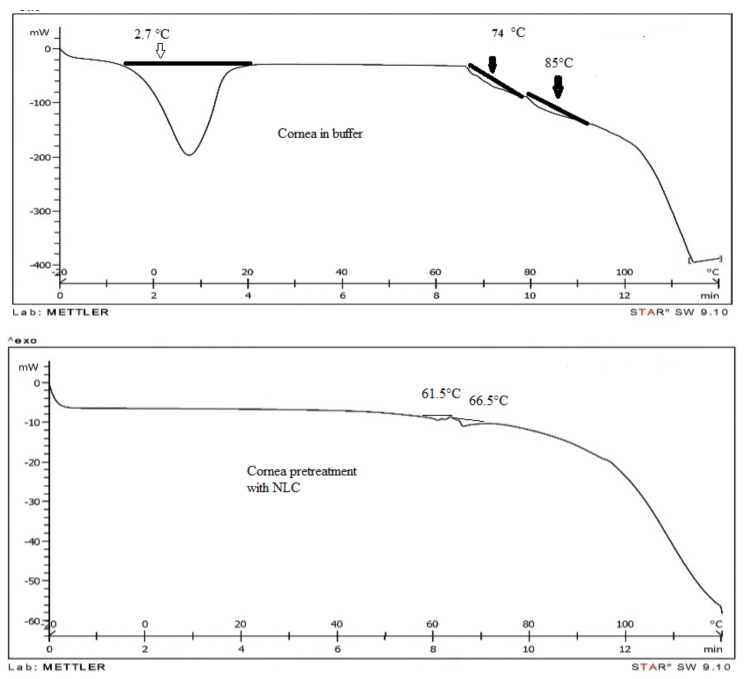

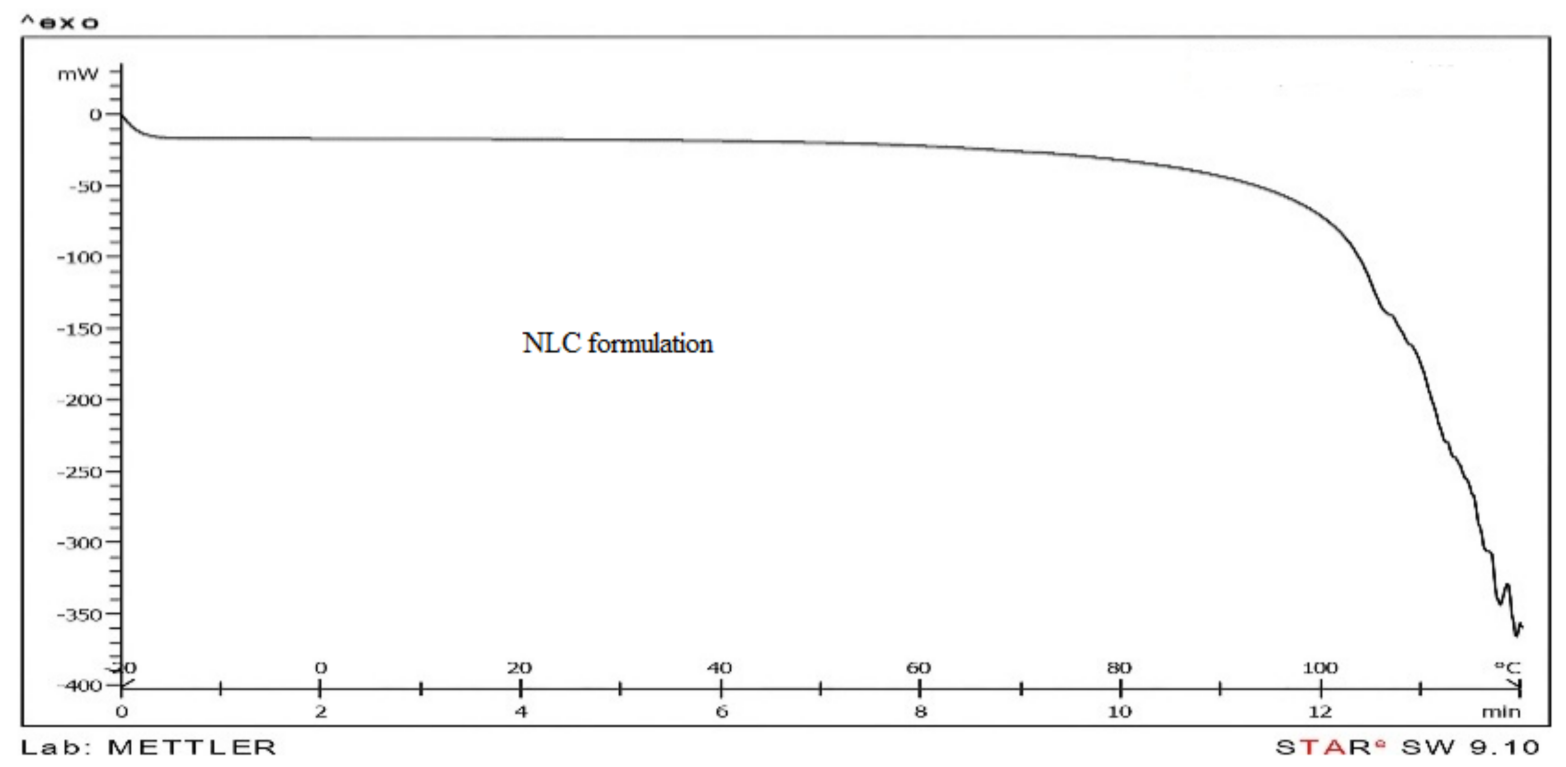
Thermograms: (**Top**) cornea in buffer solution; (**Middle**) cornea in contact with NLC formulation; (**Bottom**) NLC formulation.

In order to evaluate cornea heat behavior, thermograms were prepared by heating in temperatures ranging from −20 to 120 °C. Different thermograms were adjusted for cornea exposed to buffer, cornea in contact with NLC formulation, and NLC formulation, respectively (Figure 4).

Several parameters, such as phase transition enthalpy ∆H and phase transition temperature, were calculated.

The results illustrate that cornea has three transition phases at temperatures of 2.7, 74, and 85 °C, respectively. The transition phase was thoroughly eliminated for cornea in contact with NLC formulations at 2.7 °C. In addition, in other two-phase transitions showed negative shifts; phase transition temperatures were 12.5 and 19.5 °C, respectively. Phase transition enthalpy significantly decreased. Therefore, it seems that formulations absolutely affect cornea structure and alter phase transition. It should be mentioned that phase transition at 2.7 °C occurs due to melting free water existing in cornea [18].

As a conclusion, NLC formulations absorb free water in cornea, and due to film forming and mucoadhesion properties they result in extended existence in the ocular system. For human cornea, one transition due to collagen denaturation in 56–74 °C was reported [24]. The impact of different factors, such as surfactant, on cornea water content was also evaluated. For example, cetylpyridinium chloride as a cationic surfactant caused a decrease in cornea water content, while benzalconium chloride as a cationic surfactant had the opposite effect. It has been proven that cetylpyridinium has no impact on water binding [18]. Therefore, NLC formulation due to the presence of surfactant or other factors can decrease free water in the cornea. This effect didn’t cause any irritation in rabbit cornea, but more study is need for judgment about safety of NLC.

## 4. Discussion

NLC is the second generation of lipid nanoparticles with advantages of SLNs, while overcoming limitations such as low EE%, poor long-term stability, and possibility of drug expulsion. NLC shows great ability for ocular drug delivery due to better compatibility and modified drug release kinetics [25,26]. In the present study, propranolol-hydrochloride-loaded NLCs were prepared and characterized, and propranolol hydrochloride permeability through rabbit cornea by NLCs was evaluated. The percentage of liquid lipid in NLCs has an impact on formulation properties. An increase in liquid lipid promotes drug loading and simultaneously decreases particle size and drug release from lipid nanoparticles. Different results have been reported about impact of liquid lipid on NLC properties. Sangsen et al. indicated that increasing the amount of liquid oil increased the particle size and decreased size distribution, while curcumin entrapment efficiency and release profile were not affected by amount of liquid lipid [27]. Curcumin is lipophilic with high affinity to loading into NLCs, so an increase in liquid oil did not show any impact on EE%. However, propranolol hydrochloride is a hydrophilic compound with improvement in loading by increasing in liquid oil. In NLCs, liquid and solid lipids produce imperfection in crystal order, which causes higher drug loading by leaving enough space to accommodate drug molecules [28]. The degree of imperfection depends on the liquid oil. Oleic acid, which is a monounsaturated fatty acid form of stearic acid, produces low imperfection in crystal order [29]. It seems that the reason for the low EE% of propranolol hydrochloride is low imperfection in NLCs. On the other hand, diethyl glycol monoethylether (Transcutol) is a new enhancer which has solubilization ability apart from integration with polar or non-polar solvents. The concentration of Transcutol was 1% while used as an absorbance enhancer [21]. Transcutol increased propranolol hydrochloride solubility in NLCs, thus increasing EE%. A similar result was previously reported for didanoside-loaded in NLC as hydrophilic compound [30]. In addition, surfactant/lipid ratio has an effect on nanolipid properties; it was studied in nitrofurazone permeation through rat skin in a previous experiment [31]. In the present study, higher surfactant/lipid ratios produced smaller particle size and higher drug solubility in NLCs. This effect was reported for genistein and fluocinolone acetonide-loaded in NLCs [32,33].

Drug loading and release profile of NLCs depend on several factors such as production parameters (method, temperature, etc.), lipid and drug properties, and surfactant concentration. Propranolol hydrochloride is a water-soluble compound which has no tendency toward the lipid phase; on the other hand, the preparation method for nanoparticles is cold homogenization. The drug release profile suggested the loading model followed by solid solution and drug-enriched shell patterns. Mostly solid solution patterns occurred during cold homogenization, where the drug substance was homogenized in solid lipid matrix. Shell patterning predominated during warm homogenization, especially for hydrophilic drug substances. However, during cooling, redistribution from the aqueous phase to lipid core occurs and drug remains in the outer matrix by film formation. As cold homogenization was applied in this study and the burst effect was negligible, the solid solution pattern dominated. Drug migration towards the shell occurred during cooling, as it has high hydrophilicity, so that drug content was much higher in the outer matrix than in the core, also resulting in a drug-enriched shell pattern. We conclude that the drug release profile follows both solid solution and drug-enriched patterns.

The effect of interaction between drug and Compritol ATO 888 on the loading model and drug release through SLN has been reported for tetracaine and etomidate with low melting points and prednisolone with a high melting point [27,28]. The results showed approximately 90% loading of the tetracaine and etomidate as lipophilic compounds in SLNs. On the other hand, 20–80% of loaded tetracaine was released in6 h and particle size was introduced as a major factor influencing the release. In the latter article, particle surface area and diffusion coefficient between oily and aqueous phases were mentioned as major factors affecting burst release. Prednisolone loading and release depended on lipid type, as loading was 83.8% without any burst release for 5 weeks for the SLNs prepared with cholesterol. However, loading in SLN-containing Compritol was 80% without burst effect and 37.2% drug release during a 5-week period. The authors concluded that SLN properties strongly depended on lipid melting point; they believed that lipids with lower melting points can produce superior practical sustained release properties.

Additionally, in another study the impact of different factors (such as surfactant, preparation method, and lipid type) was studied for nitrofurazone-loaded SLN properties [31]. Nitrofurazone loading was reported at 64% and 54.1%, using sodium lauryl sulphate (SLS) and Tween 80 as surfactant, respectively. In comparison with the present study, it was observed that although propranolol hydrochloride is more hydrophilic than nitrofurazone, loading percentage was similar to that for nitrofurazone due to liquid lipid impact on nanostructures. The results demonstrated that S/L ratio has a significant impact on P parameter, while this effect was not observed by other independent variables. Therefore, although *J*_ss_ was normalized by concentration adjustment in the donor phase, permeability through cornea was affected by S/L ratio.

In terms of permeability, the results show that independent variables did not significantly relate to *T*_lag_; thus, practically prepared formulations in the present study did not show any obvious effect on shortening the time needed for approaching cornea equilibrium. In general, drug permeability through cornea includes two steps: first, partitioning or rapid distribution of drug from carrier to cornea, and second, permeation in different cornea layers. In order to determine the effects of different formulations on each step, the relation between nano-carrier properties and permeation parameters through cornea was recognized initially. The result indicates that there are no significant integrations between particle size, *J*_ss_ and *P* (*p* = 0.244). Therefore, particle size was not an important factor for *J*_ss_ and *P*, practically speaking. Since NLC formulations are not capable of *T*_lag_ alteration, we conclude that formulations did not change cornea structure and consequently had no impact on diffusion through different cornea layers.

On the other hand, since the formulations influenced P coefficient, they mainly affect drug partitioning into cornea. In other words, NLC formulations, due to their lipophilic nature, reach the inner parts of the eye. This property was also reported regarding tobramycin ocular delivery in rabbit eye, which showed that drug concentration was significantly higher in all ocular tissues after ocular and intravenous administration of tobramycin SLN formulation, with respect to reference formulations, and only tobramycin SLN allowed drug penetration into retina [14]. In addition, NLC formulations were also used for enhancing ibuprofen ocular absorption [19]. The results demonstrate that Gelucire (Gattefosse, Saint-Priest, France) as lipid and Transcutol promote drug permeation, as *P*_app_ coefficient was about 1.5 times higher than control. However, in a recent study Transcutol didn’t significantly impact ocular absorption, while NLCs increased *P* coefficient up to 20 for propranolol hydrochloride.

In conclusion, due to the hydrophilic nature of propranolol hydrochloride, its impact on *P*_app_ parameter was much higher than that of lipophilic compounds such as ibuprofen. Mucoadhesion, increased corneal retention time, and enhanced permeation due to cellular uptake by corneal epithelial cells were reported as the main reasons for ocular delivery of topical lipid nanoparticles [34]. Based on the hydrophilic property of propranolol hydrochloride and obtained results in this study, it seems that NLCs increased drug partitioning into cornea without any alteration in cornea structure.

## 5. Conclusions

In conclusion, NLC formulations improved propranolol hydrochloride as a hydrophilic compound permeation through rabbit cornea. On the other hand, this effect of nanoparticles may increase drug partitioning from carrier into cornea. Thermograms of cornea after pretreatment with NLCs demonstrated lose of free water due to surfactant. Structures responsible for transition phases were affected by NLCs. However, the effect of NLCs on permeability parameters demonstrated no structural change to cornea. It is suggested that structures belonging to transition phases are not responsible for the corneal barrier against drug permeability. Drug loading efficiency for hydrophilic compounds like propranolol hydrochloride was suitable. Drug release profile demonstrated low burst effect and a good sustained release property. Formulation (1), with the highest loading capacity, demonstrated the lowest amount of drug permeated through cornea. This finding suggests that formulation characteristics are the main factor for determination of drug permeability through rabbit cornea. Based on permeation data, Formulation (4) can be deemed the best NLC formulation that caused the highest drug permeability through cornea.

## Figures and Tables

**Table 1:** Formulation characterizations for propranolol hydrochloride nanostructure lipid carrier (NLC) according to full-factorial design and entrapment efficiency (EE%) (all concentrations are presented as *w*/*v* % and referred to total volume of suspension).

Formulation No.	State in Full Factorial Design	Drug%	%L (Oleic Acid%)	Transcutol%	Surfactant%	Compritol%	EE%
1	+++	0.5	1.5	1	3	13.5	61.23 ± 3.12
2	++−	0.5	1.5	0	3	13.5	58.13 ± 2.29
3	+−+	0.5	0.5	1	3	14.5	48.98 ± 1.72
4	−−+	0.5	0.5	1	1.5	14.5	28.53 ± 2.44
5	−+−	0.5	1.5	0	1.5	13.5	39.72 ± 3.05
6	+−−	0.5	0.5	0	3	14.5	45.11 ± 1.52
7	−++	0.5	1.5	1	1.5	13.5	40.88 ± 2.25
8	−−−	0.5	0.5	0	1.5	14.5	35.95 ± 1.47

**Table 2:** Mean particle size and polydispersity index (PDI) for propranolol hydrochloride formulations (mean ± SD, *n* = 5).

Formulation No.	State in Full Factorial Design	Particle Size (nm)	PDI
1	+++	385 ± 30	0.44 ± 0.04
2	−++	491 ± 51	0.51 ± 0.03
3	+−+	554 ± 29	0.29 ± 0.018
4	+−−	840 ± 33	0.38 ± 0.035
5	−+−	462 ± 28	0.5 ± 0.025
6	−−+	686 ± 56	0.56 ± 0.011
7	++−	706 ± 19	0.22 ± 0.02
8	−−−	880 ± 66	0.4 ± 0.03

**Table 3:** Different parameters regarding drug release from lipid nanoparticles (mean ± SD, *n* = 3). R4% and R48%: percentage of drug releaseed after 4 and 48 h.

Formulation No.	State in Full Factorial Design	R4 (%)	R48 (%)
1	+++	8.21 ± 0.37	23.38 ± 1.15
2	−++	5.4 ± 0.59	27.66 ± 1.38
3	+−+	8.45 ± 0.61	36.29 ± 1.57
4	+−−	20.71 ± 1.33	55.64 ± 4.11
5	−+−	10.4 ± 0.36	34.5 ± 3.05
6	−−+	14.5 ± 0.94	30.6 ± 1.96
7	++−	17.67 ± 1.42	42.2 ± 3.12
8	−−−	16.05 ± 0.95	41.17 ± 1.64

**Table 4:** A comparison between correlation coefficient (*r*^2^) and release rate constant (*k*) from nanostructure lipid carriers (NLCs) in different pharmacokinetic models.

Formulation No.	State in Full Factorial Design	Zero-Order Kinetic		First-Order Kinetic		Higuchi Model	
		*r* ^2^	*k*	*r* ^2^	*k*	*r* ^2^	*k*
1	+++	0.674	0.423	0.44	0.017	0.85	3.71
2	−++	0.663	0.506	0.34	0.02	0.86	4.49
3	+−+	0.776	0.708	0.39	0.024	0.92	6.04
4	+−−	0.726	0.92	0.53	0.013	0.9	7.95
5	−+−	0.88	0.69	0.51	0.027	0.96	5.65
6	−−+	0.67	0.43	0.54	0.009	0.85	3.74
7	++−	0.7	0.68	0.46	0.013	0.88	5.96
8	−−−	0.72	0.665	0.54	0.013	0.89	5.81

**Table 5:** Parameter impacts on the permeability of different formulations of propranolol hydrochloride through cornea.

Formulation No.	State in Full Factorial Design	*Q*_5_ (mg/cm^2^)	*J*_ss_ (mg/cm·s^−1^)	*T*_lag_ (h)	*P* (cm/s)
1	+++	0.7 ± 0.055	0.181 ± 0.012	0.95 ± 0.066	0.012 ± 0.002
2	−++	0.725 ± 0.038	0.178 ± 0.014	0.85 ± 0.09	0.0123 ± 0.0014
3	+−+	0.761 ± 0.062	0.185 ± 0.016	1.05 ± 0.095	0.015 ± 0.0009
4	+−−	1.705 ± 0.12	0.493 ± 0.025	0.95 ± 0.059	0.068 ± 0.001
5	−+−	1.220 ± 0.088	0.331 ± 0.014	0.88 ± 0.072	0.033 ± 0.0001
6	−−+	0.625 ± 0.055	0.155 ± 0.009	0.75 ± 0.083	0.014 ± 0.0006
7	++−	1.42 ± 0.13	0.417 ± 0.027	0.95 ± 0.047	0.041 ± 0.0008
8	−−−	0.885 ± 0.035	0.24 ± 0.017	1.10 ± 0.087	0.027 ± 0.0005
Control	−	0.229 ± 0.011	0.053 ± 0.002	0.85 ± 0.069	0.002 ± 0.00005
